# A systematic review of outcome measurement instruments used in pouch anal and vaginal fistulae: a COSMIN-based analysis

**DOI:** 10.1007/s11136-025-03911-4

**Published:** 2025-03-01

**Authors:** Easan Anand, Shivani Joshi, Lillian Reza, Kapil Sahnan, Phillip Lung, Ailsa Hart, Phil Tozer

**Affiliations:** 1 St Mark’s the National Bowel Hospital, Acton Lane, London, UK; 2 Imperial College London, Exhibition Road, London, UK

**Keywords:** Pouch Fistula, Pouch anal fistula, Pouch vaginal fistula, Patient reported outcome measures, COSMIN

## Abstract

**Purpose:**

Pouch-related fistulae are devastating complications of ileoanal pouch surgery, which is performed to improve the quality of life (QoL) for patients who have had a proctocolectomy. Their management is limited by inconsistent evidence, including using poorly and heterogeneously defined outcomes. This study aims to identify all Outcome Measurement Instruments (OMIs) used in pouch fistula research, including Patient-Reported Outcome Measures (PROMs) and Clinician-Reported Outcome Measures (ClinROMs) and evaluate their quality using COSMIN guidelines to help select the best tool for a standardised core outcome measurement set in a future consensus study.

**Methods:**

A systematic review was conducted to identify all OMIs used in ileo-anal pouch fistulae studies, from MEDLINE, Embase, and the Cochrane Library. We evaluated existing OMIs based on COSMIN guidelines and used the GRADE approach to assess evidence quality. Results were synthesized narratively.

**Results:**

Among 91 studies, 13 OMIs were reviewed. Pouch-specific instruments performed poorly in key domains of reliability, validity, and responsiveness. Only 17.6% of studies assessed QoL using PROMs. The best-performing instruments were the SF-36 and IBDQ. The Ileoanal Pouch Syndrome Severity Score was the only pouch-specific instrument that involved patients in its development and although useful for pouch dysfunction, it lacks specific QoL assessment and was not validated in pouch-fistulae patients.

**Conclusion:**

Existing OMIs for pouch-related fistulae lack adequate measurement properties, with no PROMs specifically validated for QoL in this population and very few instruments involving patients in their development. There is an unmet need for a validated PROM specifically for QoL in pouch-related fistulae.

## Plain english summary

Pouch surgery is performed to improve the quality of life of patients who have had their colon and rectum removed by avoiding a permanent stoma. Pouch-related fistulae are complications of pouch surgery that are difficult to treat, with limited high-quality evidence. The scientific literature typically emphasises outcomes such as healing or recurrent surgery without focussing on quality of life and continence. This study aimed to identify all outcome measurement instruments (OMIs), including Patient Reported Outcome Measures (PROMs) and Clinician Reported Outcome Measures (ClinROMs), used in pouch fistula research and to evaluate the quality of these instruments. There are no specific PROMs developed for pouch fistula patients, and existing tools lack patient involvement. The best instruments were the generic Short Form (SF-36) and Inflammatory Bowel Disease Questionnaires (IBDQ), that have not been tested in pouch fistula patients. There is a need for a high-quality instrument which is developed with patients specifically for pouch fistula patients.

## Introduction

### Rationale

An ileoanal pouch is an operation performed to improve the quality of life (QoL) for patients who have had a proctocolectomy (surgery to remove entire bowel and rectum) by enabling a stoma – free life through the creation of a ‘pouch’ of small bowel which acts like the native rectum, to store and expel stool [1, 2, 3]. A pouch anal or vaginal fistula is an abnormal connection between the pouch/anus and the perineum or vagina. Pouch-related fistulae are complications of surgery causing debilitating symptoms such as pain, discharge (stool or gas), infections, or recurrent abscesses, which severely diminish QoL. They often arise as a result of complications like anastomotic leaks and pelvic sepsis, with the most common types being pouch-perineal and pouch-vaginal fistulae, occurring in 4% to 16% of cases [4–6]. The development of a pouch fistula significantly increases the risk of pouch failure, when the pouch is diverted by ileostomy or removed, with up to 71% of pouch fistula patients ultimately requiring a permanent stoma [7, 8]. Pouch fistula management depends on the aetiology [9], fistula anatomy, pouch function and patient choice. Options include medical and surgical therapy [4–6]. Management of pouch-related fistulae should be patient-directed, and measurement of success should also be determined by the patient. A core outcome set (PAVFCOS) (Table 1) has been developed to standardise outcome selection in pouch-related fistula research, with QoL central to that patient-centred COS [10, 11].

**Table 1:** Pouch anal and vaginal fistula Core outcome set (PAVFCOS)

Clinician – reported outcome measures
1. Fistula healing (clinical and radiological)
2. New fistula or abscess
3. Need for rescue intervention (minor or major)

Patient – reported outcome measures
4. Pain related to fistula and surrounding area
5. Global assessment of continence
6. Impact on quality of life of fistula discharge
7. Global quality of life assessment

Only around half of existing papers on outcomes following ileoanal pouch surgery use PROMs to assess the effectiveness of treatment [12]. While generic measurement instruments exist, they often fall short in comparison to disease-specific or contextually tailored tools, such as those designed specifically for pouch-related conditions. The effectiveness of these instruments is significantly enhanced when patients are actively involved in their development and validation.

There have been no studies to date assessing the quality of outcome measurement instruments (OMIs) in pouch fistula research. COSMIN guidance on systematic reviews of PROMs has been developed to aid in the selection of measurement instruments in a core outcome measurement set using a patient-centred approach[13], and has been adapted for use in ClinROMs [14, 15]. Binary clinician-reported outcomes, like fistula healing or new fistulas/abscesses, do not require formal measurement instruments but do need careful definitions and measurement parameters, which will be addressed through the development of a core outcome measurement set.

### Objectives

**Fig. 1:**
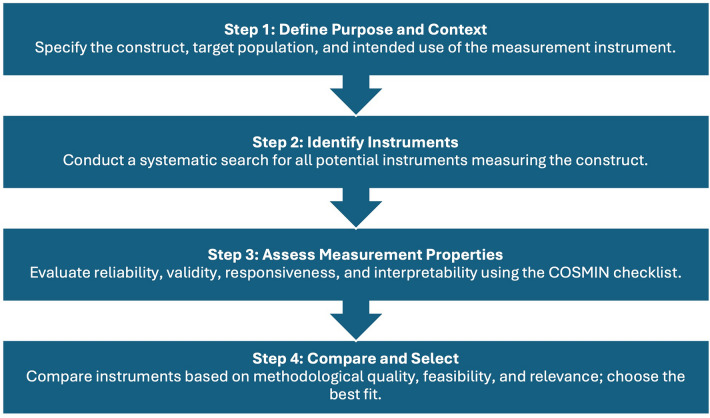
Adapted from COSMIN guideline for selecting an outcome measurement instrument

The objective of this systematic review is to follow COSMIN guidance for selecting an OMI (Fig. 1), bridging the existing gap in the literature by identifying all outcome measurement instruments (OMIs) used in ileoanal pouch fistula research, representing step 2 in the selection process for a core outcome measurement set (COMS)[13]. Following identification, we will assess the quality and feasibility of these OMIs using COSMIN guidelines for systematic reviews [16, 17], step 3 in the OMI selection process for a COMS. Determining the most relevant items for inclusion in a COMS (step 4), requires a Delphi consensus involving patients and experts, and will form the focus of a future study (PAVFCOMS).

## Methods

This review was conducted according to COSMIN guidelines for systematic review of OMIs, and results reported in accordance with the PRISMA-COSMIN checklist for OMIs [15, 16, 18].

### Eligibility criteria

This review included studies involving adult patients with ileo-anal pouch fistulae following restorative proctocolectomy for ulcerative colitis (UC), familial adenomatous polyposis (FAP), Crohn’s disease (CD), or indeterminate colitis. The terminology for ileo-anal pouches varies in the literature and includes terms such as ileoanal pouch, restorative proctocolectomy, ileoanal anastomosis, ileal anal pouch-anastomosis, and ileal pouch-anal anastomosis. Similarly, pouch fistulae are referred to as pouch-vaginal fistula, pouch-related fistula, pouch anal fistula, colonic pouch fistula, and ileal pouch-anal anastomosis-related fistula. Studies involving paediatric patients are excluded. Interventions encompass all medical and surgical treatments for pouch-related fistulae, such as pouch excision, pouch revision, ileostomy formation, gracilis transposition, redo IPAA, loose seton placement, transanal ileal pouch advancement flap, transanal repair, and transvaginal repair. Eligible studies included all articles (RCTs, observational studies, case series, case reports and abstracts) without size limitations, published from 1978 onwards.

### Information sources

The OVID Medline, Embase and CENTRAL databases were searched from 1978 (the development of the ileoanal pouch) – February 2024. Grey literature searches and pouch-relevant OMIs were identified through cross-referencing the bibliographies of key review articles.

### Search strategy

A comprehensive search strategy was developed with RCS England Librarians to ensure all relevant OMIs were captured in the systematic search. The search strategy was developed using a PICO framework and included all relevant PROMs or ClinROMs used in pouch fistulae studies. MeSH terms included were “anal or rectal or perine*”, rectal or vaginal fistula, and “ileal-pouch","IPAA", “restorative proctocolectom*, “Proctocolectomy, Restorative”. The full search strategy is attached as an appendix. Snowball sampling was employed to identify in-depth validation studies for each OMI identified in the review, with a practical limit of up to three studies per instrument. However, if more than three studies were available, the selection prioritized the top three studies based on a thorough full-text review. This method allowed for the comprehensive appraisal of measurement properties within an appropriate timeframe.

### Data collection process

Titles and abstracts were screened independently by 2 authors (EA and LR) and disputes resolved through discussion with senior authors (PT and AH). The screening process and data extraction were performed using Covidence systematic review software (Veritas Health Innovation, https://www.covidence.org). A COSMIN appraisal of OMIs was performed using pre-defined tables (https://www.cosmin.nl/finding-right-tool/conducting-systematic-review-outcome-measurement-instruments/).

### Data items

Tailored data fields were constructed to capture pertinent information from studies in pouch-related fistulae. All interventions, outcomes and measurement instruments used were systematically extracted and synthesized. Study characteristics of OMI development and validation studies, measurement properties, feasibility and interpretability were recorded using standardised COSMIN reporting templates [19]. Missing information or data were recorded as a blank cell on any relevant table, per COSMIN reporting instructions.

### Study risk of bias assessment

Two reviewers (EA and SJ) independently evaluated the risk of bias using the COSMIN risk of bias tool for all OMIs used in pouch anal and vaginal fistula research [17]. Each study was assessed against COSMIN criteria to identify potential sources of bias, ensuring transparent evaluation of the OMIs’ measurement properties. Disagreements were resolved through discussion with senior authors (AH and PT).

### Measurement properties

**Fig. 2:**
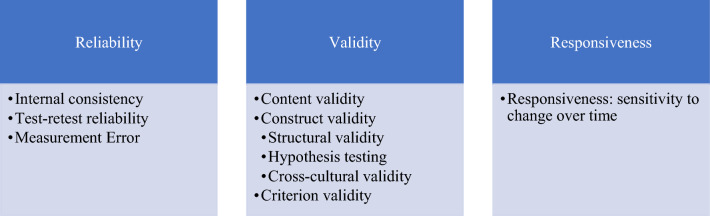
The COSMIN taxonomy: measurement properties of outcome measurement instruments (OMI) (Adapted)

Two reviewers (EA and SJ) independently evaluated the psychometric properties of each OMI development and/or validation studies using the COSMIN checklist [15]. This checklist provides a comprehensive framework for evaluating PROMs and has been adapted for use in ClinROMs evaluating clinician-led measurement tools. Each identified OMI was systematically evaluated against COSMIN criteria across three key domains of reliability, validity and responsiveness (Fig. 2**).**

Discrepancies were resolved through discussion with senior authors (AH and PT).

### Synthesis methods

The diverse nature of the studies including variations in study design, patient populations, and outcome measures precluded mixed effects modelling. In light of the limited availability of high-quality trials utilizing OMIs, a qualitative summary was adopted for this systematic review, as suggested in the COSMIN user manual [16, 17]. Findings were incorporated from the assessment of measurement properties and methodological quality of included studies to highlight the strengths, limitations and applicability of each OMI within the clinical context of pouch fistula research and identify gaps for future research in pouch-related fistulae management.

### Certainty assessment

The strength of evidence for the use of OMIs in pouch fistula patients was assessed and assigned utilizing the GRADE (Grading of Recommendations, Assessment, Development, and Evaluation) system, a widely recognized framework for evaluating the quality of evidence and strength of recommendations in systematic reviews and meta-analyses [20], and incorporated into the COSMIN user manual for systematic reviews of OMIs [13, 16, 17].

## Results

### Study selection

**Fig. 3:**
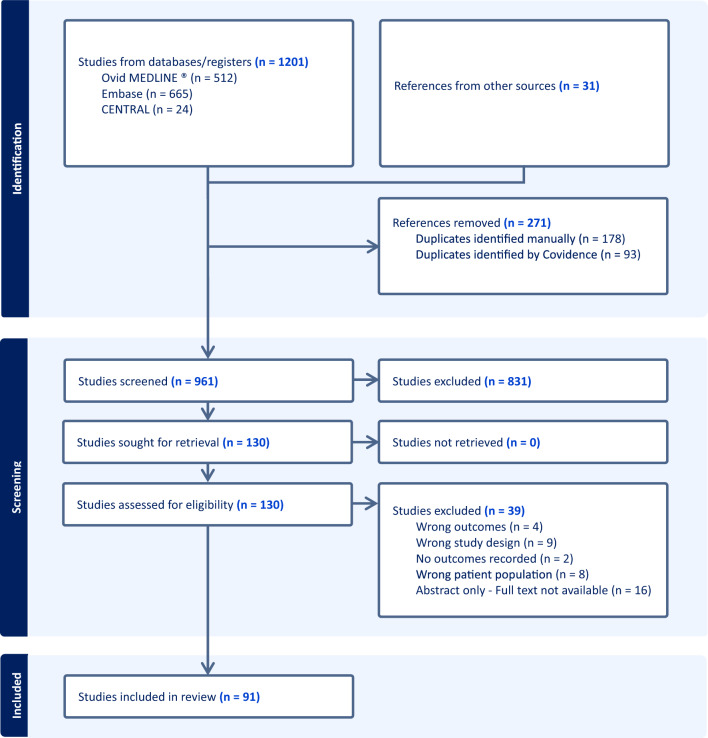
PRISMA flow diagram

The study selection process for the systematic review of OMIs was conducted following COSMIN guidelines (PRISMA flow diagram Fig. 3). A comprehensive search of electronic databases was performed, yielding a total of 1,201 records. After removal of 271 duplicates, 961 unique studies remained. These records underwent preliminary screening by two authors independently (EA and LR) based on titles and abstracts, resulting in the exclusion of 831 records that did not meet inclusion criteria. During the full-text review of 130 articles, 39 articles were excluded due to reasons such as not focusing on PROMs, not involving pouch-related fistulae patients, or lack of full text availability. Ultimately, 91 studies met the inclusion criteria and were included in the systematic review (Appendix: Table 8).

### Study characteristics

Of 91 studies selected for full text review, most were observational cohort studies or case series, whilst half of the research (52 out of 91 studies) was conducted in the UK or US. A significant number (74 out of 91) focused on the outcomes of surgical interventions, with a recent trend towards utilizing stem cell therapy, observed in 6 out of the 91 studies (Appendix: Table 1).

The mean age of patients, where reported, was 40 years (SD 5.9), and there was a predominance of female participants (n = 1130, 80.8%). Among the 4176 patients captured, 835 patients had pouch vaginal fistulae, compared to 258 cases of pouch perineal or anal fistulae. The remainder were non-fistulising complications of pouch surgery. When reported, anastomotic leak or pelvic sepsis were leading causes of pouch-related fistulae, accounting for 271 patients, closely followed by CD-related fistulae in 210 patients. Historically assessment of fistula aetiology was often flawed, with studies using the presence of a fistula as de facto evidence of CD [9]. A complete analysis of documented fistula aetiology is presented in Appendix: Table 2.

### OMI characteristics

The outcomes assessed in studies were aligned with each of the 7 items from the core outcome set (Table 1) [11]. Patient reported outcomes include pain, continence and quality of life. Clinician – reported outcomes were binary and include fistula healing, new fistula or abscess and the need for rescue intervention.

Table 2 provides an overview of the 13 OMIs used in 91 studies identified in the two-part systematic search, divided into ClinROMs and PROMs. Each OMI identified assesses at least one of the outcomes outlined in the core outcome set. The **CCFPPQ** and **OPF** assess multiple outcomes including continence and quality of life. Of 91 pouch-related fistula studies, only 16/91 (17.6%) assessed quality of life (Appendix: Table 3). Formal assessment tools include the **CQGL** in 7/91 (7.7%), the Mayo/Generic subjective scale in 5/91 (5.5%), the **IBDQ** in 2/91 (2.2%), **GIQLI** in 1/91 (1%) and the **SF-36** in 1/91 (1%).

**Table 2:** Study summary of all 13 Pouch—related outcome measurement instruments (OMIs) identified in the systematic review process and literature search (n = number of articles using PROM in a pouch fistula related study)

	n	KEY PAPER	Core outcomes assessed
*ClinROMs (Clinician reported outcome measures)*			
Cleveland Clinic Foundation Pelvic Pouch Questionnaire (CCFPPQ)	3	Kirat et al. [36]	Global assessment of continence Impact on quality of life of fistula discharge Global quality of life assessment
Oresland pouch function (OPF)	1	Oresland et al. [1]	Global assessment of continence Impact on quality of life of fistula discharge Global quality of life assessment
Pouchitis Disease Activity Index (PouchitisDAI)	5	Sandborn et al. [2]	Fistula healing
modified Pouchitis disease activity index (mPDAI)	1	Shen et al. [21]	Fistula healing
Perianal Disease Activity Index (PDAI)	1	Irvine [37]	Fistula healing
*PROMs (Patient reported outcome measures)*			
Jorge & Wexner incontinence grading score (WI)	1	Jorge and Wexner [33]	Global assessment of continence
Cleveland Global Quality of Life Score (CGQL)	5	Fazio et al. [38]	Global quality of life assessment
Gastrointestinal Quality of Life Index (GIQL)	1	Eypasch et al. [34]	Global quality of life assessment
Inflammatory Bowel Disease Questionnaire (IBDQ)	2	Guyatt et al. [30]	Global quality of life assessment
Short Form 36 (SF-36)	1	Ware et al. [31]	Global quality of life assessment
*Pouch specific ClinROMs or PROMs*			
Pouch Functional Score (PFS)	0	Lovegrove et al. [39]	Need for rescue intervention Global assessment of continence
Ileoanal Pouch Syndrome Severity Score (IPSSS)	0	Cavallaro et al. [32]	Global assessment of continence Global quality of life assessment
Pouch Dysfunction Score (PDS)	0	Brandsborg [40]	Global assessment of continence

Continence was assessed subjectively by the clinician in 30/91 studies (33%), and objectively using the **CCFPPQ** in 5/91 (5.5%) and the **WI** scale in 4/91 (4.4%). Fistula healing was predominantly assessed clinically in 76 studies (83.5%), although there was sparing use of ClinROMs including the **PouchitisDAI** (5/91, 5.5%), **mPDAI** (1/91, 1%) and the **PDAI** (1/91, 1%).

Three pouch-specific OMIs were identified: the **PFS**, the **IPSSS**, and the **PDS**. None of these instruments have been used in clinical trials assessing treatment for pouch-related fistulae or validated in fistula patients.

### Characteristics of individual PROMS

#### The characteristics of the 13 OMIs used in studies on pouch fistulae are outlined in Table 3

**Table 3:** Characteristics of individual OMIs

OMI* (reference to first article)	Construct(s)	Target population	Mode of administration (e.g. self-report, interview-based, parent/proxy report etc.)	Recall period	(Sub)scale (s) (number of items)	Response options	Range of scores/scoring	Original language	Available translations	Patient involvement in development study
*ClinROMs*										
CCFPPQ	Pouch Function	IPAA	Self-reported	NA	9	Bowel frequency (number of bowel movements per 24 h), urgency (inability to defer bowel movements for more than 15 min), faecal incontinence (inadvertent passage of liquid or solid stool), stool seepage (soiling during day or night), use of pads, and dietary, social, work, and sexual restrictions	Continuous	English	English	No
OPF	Pouch Function	IPAA	Self-reported	2 Weeks/ Bowel diary	9	Bowel frequency (day/night), Urgency, Evacuation difficulties, Soiling/Seepage, Perianal soreness, Protective pad, Dietary restrictions, Medication, Social handicap	0 (Good function) - 15 (Poor function)	Swedish	English	No
PouchitisDAI	Pouchitis	IPAA	Clinician	NA	7	Clinical/Endoscopic inflammation/Histological inflammation	0 - 18 (> 7 defined as clinically significant pouchitis)	English	English	No
mPDAI	Pouchitis	IPAA	Clinician	NA	6	Clinical and Endoscopic	0 - 12 (> 5 as clinically significant)	English	English	No
PDAI	Perianal Crohn's disease	pCD	Self-reported/ Clinician	NA	5	Discharge, Pain, Restriction of sexual activity, Type of perianal disease, Degree of induration	0 (No disease)- 20 (Severe)	English	English	No
*PROMs*										
WI	Incontinence	All patients	Self-reported	NA	5	Solid, Liquid, Gas, Wears pad, Lifestyle alteration	0 (Perfect continence)- 20 (Complete incontinence)	English	42 translations	No
CGQL	QOL	Post- IPAA	Self-reported	NA	3	Quality of Life, Quality of Health, Energy Level	0 - 1 (Perfect QOL)	English	English, Italian	No
GIQLI	QOL	GI Disease	Self-reported	2 Weeks	36	Subjective GI QOL	0 - 144 (perfect QOL)	German	English	Yes
IBDQ	QOL	IBD	Self-reported	4 weeks	32	IBD QOL: bowel symptoms, systemic symptoms, emotional function, social function	32 (worst) - 244 (Perfect quality)	English	127 translations	Yes
SF-36	QOL	All patients	Self-reported	4 weeks	36	Functional status, Wellbeing, Overall evaluation of health	0 (Worst) - 100 (Best)	English	193 translations	Yes
*Pouch specific ClinROMs or PROMs*										
PFS	Pouch Function	IPAA	Self-reported	NA	7	24H Stool frequency, Nocturnal stool frequency, Urgency, Major incontinence, Minor incontinence, Antidiarrhoeals, Antibiotics	0 - 30 (Worse score)	English	English	No
IPSSS	Pouch function	IPAA	Self-reported	NA	3	3 Domains: 15 questions	0 - 145	English	English	Yes
PDS	Pouch dysfunction	IPAA	Self-reported	2 Weeks	6	5 Domains, 1 QOL	0 - 7.5	Danish	English	No

The **CCFPPQ** and the **OPF** questionnaire evaluated pouch function covering aspects such as bowel frequency, urgency, stool seepage, and incontinence. Neither of these OMIs involved patients during their development, and information regarding their creation was limited. The **PouchitisDAI**, which initially included clinical, endoscopic, and histological criteria for diagnosing pouchitis, was modified to remove histological assessment in the **mPDAI**. Continence was assessed (in part) by both the **CCFPPQ** and the **WI**. The **CQGL**, specifically designed for pouch patients, assessed global quality of life in pouch patients. **GIQLI** and **IBDQ** were QOL scales developed for populations with gastrointestinal and inflammatory bowel disease respectively. The **SF-36** was developed as a generic health QOL scale. The **PFS**, the **IPSSS**, and the **PDS** were designed to assess pouch function and dysfunction, but none was developed specifically in patients with pouch-related fistulae.

The only OMIs to involve patients in the original development study were the **GIQLI**, **IBDQ**, **SF-36** and **IPSSS**.

Details of the study populations in individual OMI development studies are provided in Appendix: Table 4**.**

### Interpretability and feasibility of OMI

Of the 5 indices used to assess clinical response, only the** PouchitisDAI** and **mPDAI** provide specific figures in their development study to aid in the interpretation of overall scores. A score of > 7 on the **PouchitisDAI** and > 5 on the **mPDAI **have arbitrarily been used to diagnose pouchitis [2, 21]. A > 3 point reduction in the **Pouchitis DAI **score has been used in a number of studies to define response to treatment [22]. A **PDAI** score of > 4 is suggestive of an active perianal fistula and has been used in several clinical trials, but is not specific to pouch-related fistulae [23–25]. The minimal clinically important difference is less clear, although a > 3 point change in the **PDAI **has been used signify clinical change in the assessment of perianal disease [22, 26, 27]. No index was designed to aid in the diagnosis and/or treatment of a pouch related fistula and there was no information available on score ranges, floor or ceiling effects and minimal important change levels for any of the remaining OMIs studied. An interpretability and feasibility assessment on each OMI was performed using COSMIN criteria and the results are displayed in Appendix: Tables 5 and 6**.**

### COSMIN appraisal of OMI development

The quality of the PROM development study was assessed using COSMIN criteria based on general design requirements and the strength of any cognitive interview studies reported. The majority of OMIs included had clear design constructs and context for their use, resulting in ‘Very Good’ ratings for much of general design requirements. The **IPSSS**, designed to score pouch dysfunction (rather than pouch-related fistulae) was the only OMI rated as adequate based on an excellent PROM design and patient involvement throughout its development. The remaining PROMs used in pouch fistula research were either deemed doubtful or inadequate, often as a result of complete absence of patient involvement in the development study (noted as a lack of cognitive interview study), or underreporting of essential criteria such as the comprehensibility of a questionnaire, rendering doubtful or inadequate ratings. The complete analysis is presented in Table 4.

**Table 4:** Quality of the OMI development

	OMI		PROM design							Cognitive interview (CI) study^2^			TOTAL PROM DEVELOPMENT
			General design requirements					Concept elicitation^1^	Total PROM design	General design requirements	Comprehensibility	Total CI study	
Reference		Language in which the PROM was developed	Clear construct	Clear origin of construct	Clear target population for which the PROM was developed	Clear context of use	PROM developed in sample representing the target population			CI study performed in sample representing the target population			
Fazio et al. [28]	CCFPPQ	English	V	D	V	V	A	I	I			I	I
Oresland et al. [1]	OPF	English, Swedish	V	D	V	V	A	I	I			I	I
Sandborn et al. [2]	PouchitisDAI	English	V	V	V	V	A	A	A			I	I
Shen et al. [21]	mPDAI	English	V	V	V	V	V	A	A			I	I
Irvine [37]	PDAI	English	V	V	V	V	V	D	D	V	D	D	D
Jorge and Wexner [33]	WI	English	V	V	V	V	A	D	D			I	I
Fazio et al. [38]	CGQL	English	V	V	V	V	D	D	D			I	I
Eypasch et al. [34]	GIQLI	English	V	V	V	V	V	D	D	V	V	D	D
Guyatt et al. [30]	IBDQ	English	V	V	V	V	V	D	D	V	V	D	D
Ware et al. [31]	SF-36	English	V	V	V	V	V	D	D	V	V	A	D
Lovegrove et al. 39	PFS	English	V	V	V	V	V	A	A			I	I
Cavallaro et al. [32]	IPSSS	English	V	V	V	V	V	A	A	V	V	A	A
Brandsborg et al. [40 ]	PDS	English	V	V	V	V	V	D	D	V	V	D	D

*V* Very good, *A* Adequate, *D* Doubtful, *I* Inadequate, *Empty box* Not applicable

1 When the PROM was not developed in a sample representing the target population, the concept elicitation was not further rated

2 Empty cells indicate that a CI study (or part of it) was not performed

The final rating is determined by the lowest rating across all design requirements

Each OMI was assigned a rating for each design requirement by two independent assessors (EA and SJ) before consensus was reached. Scores range from inadequate (worst) to Very good (best) based on the COSMIN checklist for PROM development studies

### COSMIN Assessment of measurement properties of OMIs (Table 5)

**Table 5:** Assessment of measurement properties and Quality of the evidence for the measurement properties of OMI (GRADE Methodology)

	*CCFPPQ*		*OPF*		*PouchitisDAI*		*mPDAI*		*PDAI*		*WI*	
	OVERALL RATING	QUALITY OF EVIDENCE	OVERALL RATING	QUALITY OF EVIDENCE	OVERALL RATING	QUALITY OF EVIDENCE	OVERALL RATING	QUALITY OF EVIDENCE	OVERALL RATING	QUALITY OF EVIDENCE	OVERALL RATING	QUALITY OF EVIDENCE
	+ /− /?	High, moderate, low, very low	+ /− /?	High, moderate, low, very low	+ /− /?	High, moderate, low, very low	+ /− /?	High, moderate, low, very low	+ /− /?	High, moderate, low, very low	+ /− /?	High, moderate, low, very low
Content validity	?		?		+/−	Low	+/−	Low	+/−	Moderate	+/−	Moderate
*Relevance*	?		+/−	Very low	+	Low	+	Low	+	Moderate	+	Moderate
*Comprehensiveness*	?		-	Very low	-	Low	-	Low	?		-	Moderate
*Comprehensibility*	?		?		+	Low	+	Low	+	Moderate	+	Moderate
Structural validity	?		?		?		?		?		?	
Internal consistency	?		?		?		?		?		+	Moderate
Cross-cultural validity	?		?		?		?		+	Moderate	+	Moderate
Measurement invariance	?		?		?		?		?		?	
Reliability	?		?		?		?		-	Moderate	+	Moderate
Measurement error	?		?		?		?		?		?	
Criterion validity	?		?		+	Low	+	Low	+	Moderate	?	
Construct validity	?		?		+	Low	+	Low	+	Moderate	+	Moderate
Responsiveness	?		?		?		+	Low	+	Moderate	?	

	*CGQL*		*GIQLI*		*IBDQ*		*SF-36*	
	OVERALL RATING	QUALITY OF EVIDENCE	OVERALL RATING	QUALITY OF EVIDENCE	OVERALL RATING	QUALITY OF EVIDENCE	OVERALL RATING	QUALITY OF EVIDENCE
	+ /− /?	High, moderate, low, very low	+ /− /?	High, moderate, low, very low	+ /− /?	High, moderate, low, very low	+ /− /?	High, moderate, low, very low
Content validity	+/−	Low	+/−	Moderate	+	Moderate	+/−	Moderate
*Relevance*	+/−	Low	+	Moderate	+	Moderate	+	Moderate
*Comprehensiveness*	?		?		+ / −	Moderate	-	Moderate
*Comprehensibility*	+	Low	?		+	Moderate	+/−	Moderate
Structural validity	?		+	Moderate	?		+	Moderate
Internal consistency	+	Low	+	Moderate	+	Moderate	+	Moderate
Cross-cultural validity	+	Low	+	Moderate	+	Moderate	+	Moderate
Measurement invariance	?		?		?		?	
Reliability	+	Low	+	Moderate	+	Moderate	+	Moderate
Measurement error	?		?		?		+	Moderate
Criterion validity	?		?		?		?	
Construct validity	+	Low	+	Moderate	+	Moderate	+	Moderate
Responsiveness	+	Low	+	Moderate	+	Moderate	?	
			GIQLI upgraded in view of multiple + measurement properties		IBDQ upgraded to moderate as multiple validation studies		SF-36 upgraded to moderate as multiple validation studies	

	*PFS*		*IPSSS*		*PDS*	
	OVERALL RATING	QUALITY OF EVIDENCE	OVERALL RATING	QUALITY OF EVIDENCE	OVERALL RATING	QUALITY OF EVIDENCE
	+ /− /?	High, moderate, low, very low	+ /− /?	High, moderate, low, very low	+ /− /?	High, moderate, low, very low
Content validity	+/−	Low	+	Moderate	+/−	Low
*Relevance*	+	Low	+	Moderate	+	Low
*Comprehensiveness*	-	Low	+	Moderate	+/−	Low
*Comprehensibility*	?		+	Moderate	+	Low
Structural validity	?		?		?	
Internal consistency	?		?		?	
Cross-cultural validity	?		+	Moderate	?	
Measurement invariance	?		?		?	
Reliability	?		+	Moderate	+	Low
Measurement error	?		?		?	
Criterion validity	?		+	Moderate	+	Low
Construct validity	?		+	Moderate	?	
Responsiveness	?		?		?	
IPSSS downgraded to moderate in view of limited measurement properties						

Each overall rating result is rated as either sufficient ( +), insufficient (–), inconsistent ( ±), or indeterminate (?). Sufficient ratings are highlighted with a green +

The quality of the evidence is graded (high, moderate, low, very low evidence), using a modified GRADE approach. High and moderate ratings are colour -coded GREEN, low and very low levels of evidence are colour—coded RED

A GRADE level is not applied if the summarised result is indeterminate (?)

The COSMIN checklist was used to assess the measurement properties of individual OMIs by two independent reviewers (EA and SJ). Each measurement property was given a final consensus rating, and where this was not possible conflicts were resolved through discussion with senior authors (PT and AH).

#### Content validity (assessed separately as per COSMIN guidelines)

The development study of the OMI, along with any content validity studies, was independently evaluated to assess its relevance, comprehensiveness, and comprehensibility **(**Table 4**).** A final content validity rating was determined through a consensus process, integrating findings from the validity studies and reviewer assessments.

The **CCFPPQ** and **OPF** provided minimal or no description of their development methodologies and were therefore assigned indeterminate ratings for content validity. Among the ClinROMs, the **PouchitisDAI**, **mPDAI**, and **PDAI** demonstrated sufficient relevance and comprehensibility but lacked evidence to support comprehensiveness. Of the generic PROMs, **CGQL** was deemed comprehensible due to its simplicity but lacked sufficient descriptions to assess relevance and comprehensiveness. The **GIQLI**, **IBDQ**, and SF-36 received sufficient ratings for relevance and showed good face validity, however, they failed to demonstrate evidence of data saturation, resulting in insufficient ratings for comprehensiveness. Of the pouch-specific PROMs/ClinROMs, the **IPSSS** was the only instrument to achieve clear and sufficient ratings across all aspects of content validity.

#### Remaining measurement properties

Each OMI development study and relevant validation studies was independently assessed for the presence of measurement properties according to the COSMIN checklist. Structural validity could only be assessed for the **SF-36** and **GIQLI**, as validation studies for these instruments included factor analysis, resulting in a sufficient rating for this property. No other OMIs reported analyses that allowed for an evaluation of structural validity. None of the ClinROMs or pouch-specific PROMs reported data on internal consistency. Among the general PROMs, the **CGQL**, **GIQLI**, **IBDQ**, and **SF-36** received sufficient ratings for this property. The **PDAI**, **WI**, **CGQL**, **GIQLI**, **IBDQ**, **SF-36** and **IPSSS** were rated as sufficient when assessing cross-cultural validity based on assessment of their development and validation studies. Among the ClinROMs, the **PDAI** was rated as insufficient for test–retest reliability, while the **WI** received a sufficient rating. All generic PROMs, along with the **IPSSS** and **PDS**, were rated as sufficiently reliable based on test–retest reliability assessments. The **SF-36** was the only OMI rated sufficient for assessing measurement error. Of the ClinROMs, the **PouchitisDAI**, **mPDAI** and **PDAI** were given a sufficient rating for criterion validity, based on correlation with a gold standard. Of the generic PROMs and pouch – PROMs, the **IPSSS** and **PDS** received a sufficient rating for criterion validity. Among the ClinROMs, the **PouchitisDAI**, **mPDAI**, and **PDAI** received sufficient ratings for construct validity. All generic PROMs were also rated as sufficient in this property. The **IPSSS** was the only pouch-specific PROM to achieve a sufficient rating for construct validity. The **mPDAI**, **PDAI**, **CGQL**, **GIQLI**, **IBDQ** all demonstrated features of responsiveness to be awarded a sufficient rating.

### COSMIN risk of bias in studies (Table 6) and quality of the evidence for measurement properties (Table 5)

**Table 6:** Quality of studies on measurement properties of OMI – risk of bias ratings

PROM	Content validity							Structural validity	Internal consistency	Cross-cultural validity	Reliability	Measurement error	Criterion validity	Construct validity		Responsiveness			
	Asking patients				Asking experts														
	Relevance		Comprehensiveness	Comprehensibility	Relevance	Comprehensiveness								Convergent validity	Known groups validity	Comparison with gold standard	Comparison with other instruments	Comparison between subgroups	Comparison before and after intervention
CCFPPQ														D	D		D	A	A
OPF																			
PouchitisDAI					D	D								I	A				
mPDAI					D	D							A	A	A				A
PDAI		D	D	D	A	A	I		I	I	A	I	I	I	I	I	A	I	A
WI					D	D													
CGQL					D	D			V				A						
GIQLI		D	D	D	D	D			D		D			I	D				A
IBDQ		D	D	D	D	D					I			I		V			
SF-36		A	A	A	A	A	V		A		A		V	I	D				
PFS														I	D			I	
IPSSS		D	D	A	V	V					A		V	D	V				
PDS		D	D	D	D	D					V		V						

*V* Very good, *A* Adequate, *D* Doubtful, *I* Inadequate, *Empty box* Not applicable

The COSMIN risk of bias checklist was used to assess the overall quality of the evidence for measurement properties of all OMIs identified, based on assessment of the initial index development study and validation studies. To enhance clarity, the risk of bias assessment for each OMI index study is presented in Table 6, as this was considered the key indicator of the quality of OMI development. The absence of ratings for a large number of studies included highlights the lack of adequate psychometric testing and many studies were given a doubtful or inadequate rating. Each study has been assigned a level of evidence according to the GRADE methodology based on the likely risk of bias in development and validation studies [20]. The level of evidence was downgraded by the presence of doubtful or inadequate studies as highlighted in Table 5. A large number of studies with an overall rating of indeterminate (?) were not assigned an overall level of evidence in line with COSMIN guidance.

The highest level of evidence using the GRADE methodology of the ClinROMs was the widely used **PDAI** which displayed sufficient content validity, construct validity and responsiveness with adequate numbers of high quality studies to be assigned a moderate level of evidence. An overall level of evidence was assigned to each OMI, provided at least one measurement property was assessed, to streamline relevance for inclusion in a core measurement set— a slight deviation from COSMIN guidance, which recommends evaluating each measurement property individually.

Of all PROMs identified in the review, the **WI** to assess incontinence, the generic QOL scales **GIQLI**, **IBDQ**, **SF-36** and the pouch dysfunction **IPSSS** were given a moderate level of evidence. Importantly, the **WI**, **SF-36**, **IBDQ** or **GIQLI** were comparable in their measurement properties to assess quality of life in a population of patients with gastrointestinal disease, but none have been validated in pouch fistula patients. The **IPSSS** was the only one of three pouch specific instruments identified that can be assigned a moderate level of evidence. However, it has not been externally validated and was not designed for use in pouch fistulae patients (instead it assesses pouch dysfunction which results in a different set of symptoms).

### Narrative synthesis of results

This COSMIN appraisal of OMIs in the context of pouch-related fistulae highlights several key findings and gaps in the current research landscape. Notably, there are no PROMs specifically designed to address pouch-fistula complications, highlighting a critical gap in tools that capture the unique challenges faced by pouch patients with fistulae.

Three pouch-specific OMIs were identified, of which there was one PROM, the **IPSSS**. This index, which assesses pouch dysfunction and pouchitis, is relatively novel and exhibits adequate measurement properties, and achieved sufficient ratings for content validity, criterion validity and construct validity. Responsiveness was not assessed in the development study. However, its interpretability and utility are constrained by the lack of external validation studies and real-world implementation, and it was not designed for use in pouch fistulae patients. The most frequently used scores, **CCFPPQ** and **CQGL** did not involve patients in their development studies and therefore did not score highly in the domain of content validity. The **CCFPPQ** was limited in other domains, predominantly because of the lack of psychometric testing and PROM development study. The **CQGL** scale and **CCFPPQ** were developed specifically for use in the ileoanal pouch population but received largely indeterminate ratings due to limited evidence available to adequately assess the quality of PROM development and measurement properties using the COSMIN checklist [28, 29].

The** PouchitisDAI** and **mPDAI** are hybrid ClinROM tools incorporating both clinician and patient-reported elements [2, 21]. These indices have been utilized for the last twenty years and demonstrate a reasonable range of measurement properties. Despite the low level of evidence supporting their use in research and clinical practice, they remain in use due to the absence of alternative tools for assessing clinical, endoscopic, and histological outcomes in this patient population.

Other PROMs identified through this systematic review were originally developed for inflammatory bowel disease (IBD) populations or general populations. Among these, the **WI** possesses adequate measurement properties and has been translated into 42 languages with numerous external validation studies, but none included pouch patients. The **SF-36** and the **IBDQ** are validated for assessing quality of life in general and IBD populations, respectively, but not pouch patients [30, 31].

## Discussion

### General interpretation & summary of findings

This systematic review provides a comprehensive evaluation of OMIs, encompassing ClinROMs and PROMs, used in the context of pouch-related fistulae, marking the first such effort to critically appraise all outcome measurement instruments employed in this population.

The main findings reveal a lack of high-quality OMIs designed to assess outcomes following interventions for pouch fistulae. Utilizing the rigorous COSMIN guidelines and risk of bias checklist, a detailed critical appraisal and analysis of the OMIs currently in use was undertaken, highlighting significant gaps in the available tools. Among the identified instruments, the **IPSSS** stands out as a pouch-specific OMI that involved patients throughout its development [32]. Although relatively novel, it demonstrates adequate measurement properties for assessing pouch dysfunction and pouchitis. It has not been validated in pouch fistula patients and does not specifically address quality of life in this group of patients.

Based on the findings of this systematic review, several inferences can be drawn regarding the use of OMIs in pouch-related fistula research. For a 'global assessment of continence,' the **WI** scale demonstrates adequate measurement properties and has been assigned a moderate level of evidence; however, its recommendation is contingent upon future validation in a pouch-fistula cohort [33]. No existing OMI adequately assessed 'pain related to fistula' or the 'impact on quality of life of pouch-related fistulae' based on current evidence. The outcomes of 'fistula healing,' 'new fistula or abscess,' and 'need for rescue intervention' are binary and require precise definitions, which can be effectively established using Delphi methodology. The **PouchitisDAI** shows adequate ratings for content and criterion validity when assessing pouch function. However, its development lacked patient involvement, resulting in lower quality of evidence and applicability. Additionally, concerns remain regarding its cost and feasibility, given the need for endoscopic and histological assessments [2]. When evaluating 'global quality of life,' both the **GIQLI** and the **IBDQ** demonstrate adequate measurement properties and have been assigned a moderate level of evidence. However, neither was developed for, nor validated in, pouch fistula patients [30, 34].

None of the existing OMIs identified were specifically developed for patients with ileoanal pouches, and many of the questions within these instruments are not directly relevant to this patient population. The pouch – specific **CCFPPQ** and **CGQL** were rated as inadequate based on their development studies and received indeterminate ratings for several measurement properties due to the lack of high-quality evidence in the literature [28, 29]. This underscores the urgent need for the development of specific OMIs tailored to the unique needs and experiences of patients with ileoanal pouches and fistulae.

### Limitations of the evidence included

There is a notable lack of research specifically targeting OMIs in ileoanal pouch patients, and none addressing pouch fistula patients. The existing heterogeneity in the literature and lack of targeted research severely limits the availability of robust and relevant data for comprehensive analysis. Furthermore, the development of OMIs for ileoanal pouches has been insufficient, with existing instruments lacking relevance and specificity to this patient population. Additionally, the process of OMI development has frequently neglected patient involvement, leading to tools that may not fully capture patient experiences and outcomes.

There are other notable indices such as the St Mark’s incontinence score (a modification of the **WI**) that may be considered for assessing continence in this population of patients, although it was not captured in this search strategy as it has not been used in pouch fistula research, neither was it developed for this unique population [35].

The specific symptoms of a pouch fistula, such as pain and discharge from the fistula site, and the unique additional impact of the fistula on quality of life are not adequately addressed by generic quality of life PROMs. Although the **IBDQ** has strong measurement properties and is widely validated, it is not specific to pouch patients or pouch-related fistulae [30]. Additionally, the impairment of continence caused by a pouch-related fistula, which may include flatus, liquid, and solid discharge from the fistula site as well as anal discharge, is another subtlety not captured by the **WI** scale.

### Limitations of the review processes

The systematic review process in ileoanal pouch fistulae research is hindered by several significant limitations. Many of the indices used in this field were developed decades ago without substantial patient involvement, which undermines their relevance and sensitivity to patient experiences and outcomes, and they often lack the rigorous psychometric testing required to establish them as high-quality measurement tools. This lack of patient input means that these indices may not fully capture the nuances and specific challenges faced by individuals with ileoanal pouches and fistulae. As a result, their validity, reliability, and overall measurement properties may be inadequate for contemporary research and clinical practice. The lack of patient-centred OMIs limits the ability to accurately assess and compare outcomes, thereby constraining the overall quality and applicability of the systematic review.

A limitation of our review is the absence of a dedicated PROM filter in the search strategy, which may have identified additional studies specifically assessing the measurement properties of OMIs identified in the review, although undertaken in other disease populations. Instead, we relied on a comprehensive search strategy focused on identifying all pouch-related fistula studies, supplemented by a snowball sampling approach to capture relevant validation studies of OMIs. While this approach ensured feasibility, it may have led to the exclusion of some measurement property studies not directly linked to pouch-related fistulae in the indexing (and therefore not relevant to this work).

Further, a review of the literature alone cannot hope to identify the measurement instruments to be used in pouch fistula research. Rather, a consensus process involving all stakeholders and, in particular, patients is needed – the development of a Core Outcome Measurement Set (COMS) – which is underway, and which this review informs.

### Implications for practice, policy and research

The findings of this systematic review of OMIs in pouch-related fistula research underscore several crucial implications for clinical practice and research. The limitations identified in existing tools highlight the importance of adopting a more patient-centred approach in the development and utilization of OMIs. The development and validation of a new PROM specific to pouch fistula patients that engages patients in the development process and undergoes rigorous psychometric evaluation is required and is under construction. There is a pressing need for longitudinal studies that assess the responsiveness of PROMs to changes in disease status and treatment interventions over time. By addressing these research gaps and developing robust and relevant PROMs, clinicians and researchers can better understand the complex challenges faced by individuals with ileoanal pouch fistulae, and crucially, improve the quality, consistency and comparability of pouch fistula research.

## Data Availability

The full search strategy is supplied as an appendix.

## Code availability

The full search strategy is supplied as an appendix.

## Glossary

**OMI**: Outcome Measurement Instrument (includes ClinROMs and PROMs)
**ClinROM:**: Clinician Reported Outcome Measure
**PROM:**: Patient Reported Outcome Measure
**CCFPPQ:**: Cleveland Clinic Pelvic Pouch Questionnaire
**OPF:**: Oresland Pouch Function
**PouchitisDAI:**: Pouchitis Disease Activity Index
**mPDAI:**: Modified Pouchitis Disease Activity Index
**PDAI:**: Perianal Disease Activity Index
**WI:**: Wexner incontinence grading score
**CQGL:**: Cleveland Global Quality of Life Score
**GIQLI:**: Gastrointestinal Quality of Life Index
**IBDQ:**: Inflammatory Bowel Disease Questionnaire
**SF-36:**: Short-Form 36
**PFS:**: Pouch Functional Score
**IPSSS:**: Ileoanal Pouch Syndrome Severity Score
**PDS:**: Pouch Dysfunction Score
